# Time-resolved analysis of *Staphylococcus aureus* invading the endothelial barrier

**DOI:** 10.1080/21505594.2020.1844418

**Published:** 2020-11-22

**Authors:** Elisa J.M. Raineri, Harita Yedavally, Anna Salvati, Jan Maarten van Dijl

**Affiliations:** a Department of Medical Microbiology, University of Groningen, University Medical Center Groningen, Groningen, The Netherlands; b Department of Nanomedicine and Drug Targeting, Groningen Research Institute of Pharmacy, University of Groningen, Groningen, The Netherlands

**Keywords:** *Staphylococcus aureus*, MRSA, endothelium, invasion, intracellular

## Abstract

*Staphylococcus aureus* is a leading cause of infections world-wide. Once this pathogen has reached the bloodstream, it can invade different parts of the human body by crossing the endothelial barrier. Infected endothelial cells may be lysed by bacterial products, but the bacteria may also persist intracellularly, where they are difficult to eradicate with antibiotics and cause relapses of infection. Our present study was aimed at investigating the fate of methicillin resistant *S. aureus* (MRSA) isolates of the USA300 lineage with different epidemiological origin inside endothelial cells. To this end, we established two *in vitro* infection models based on primary human umbilical vein endothelial cells (HUVEC), which mimic conditions of the endothelium when infection occurs. For comparison, the laboratory strain *S. aureus* HG001 was used. As shown by flow cytometry and fluorescence- or electron microscopy, differentiation of HUVEC into a cell barrier with cell-cell junctions sets limits to the rates of bacterial internalization, the numbers of internalized bacteria, the percentage of infected cells, and long-term intracellular bacterial survival. Clear strain-specific differences were observed with the HG001 strain infecting the highest numbers of HUVEC and displaying the longest intracellular persistence, whereas the MRSA strains reproduced faster intracellularly. Nonetheless, all internalized bacteria remained confined in membrane-enclosed LAMP-1-positive lysosomal or vacuolar compartments. Once internalized, the bacteria had a higher propensity to persist within the differentiated endothelial cell barrier, probably because internalization of lower numbers of bacteria was less toxic. Altogether, our findings imply that intact endothelial barriers are more likely to sustain persistent intracellular infection.

## Introduction

*S. aureus* is an important pathogen that can persist for long periods of time in the human host, be it on the skin, the mucosa or intracellularly. For most individuals, the carriage of *S. aureus* is asymptomatic and without major consequence. However, upon trauma, surgery or inadequate protection by the immune defenses, *S. aureus* may invade the human body, reach the blood stream and cause serious diseases that range from bacteremia to sepsis, endocarditis and necrotizing pneumonia. The treatment of invasive *S. aureus* infections has always been a challenge due to the pathogen’s ability to invade different phagocytic and non-phagocytic host cells, and to form thick biofilms that represent a protective niche against antibiotic therapy. Therapeutic success is nowadays also compromised as *S. aureus* has acquired resistance to many different antibiotics [1–3]. Moreover, the diversity within the species *S. aureus* is enormous, which is reflected in the different epidemiological features and pathogenicity of the clonal *S. aureus* lineages that we know today [4–8].

Among the antibiotic resistant lineages, the methicillin-resistant *S. aureus* (MRSA) has become notorious as it is associated with high morbidity and mortality [9]. MRSA first emerged in nosocomial settings, in particular causing invasive blood stream infections [10,11]. However, in recent years MRSA infections are not only caused by hospital-associated (HA) lineages, but also by community-associated (CA) lineages that affect healthy individuals with no apparent hospital contact. Contrary to HA-MRSA, the CA-MRSA lineages tend to cause soft tissue infections or more invasive infections such as pneumonia and osteomyelitis [11].

Once *S. aureus* has gained access to the blood stream, in immunocompetent healthy individuals it is usually cleared by phagocytic immune cells. However, some bacteria may survive phagocytosis, disseminate in the blood, and enter the endothelium. The endothelium has a key role in the human body as this monolayer of cells represents a barrier that can selectively control the passage, from both the apical and basal sides, of solutes, plasma proteins, immune cells (e.g. leukocytes), viruses and bacteria. This control is achieved by the activation of specific pathways and expression of different sets of proteins on the apical and the basal cell sides, and through the coordinated opening and closure of cell-cell junctions, such as tight junctions and adherens junctions. Attached to these junctions are a variety of adhesion molecules, including the ‘cluster of differentiation 31ʹ (CD31) protein (also known as ‘platelet endothelial cell adhesion molecule-1ʹ [PECAM-1]), which provide connections to the actin cytoskeleton of the cells [12]. These structures can be disrupted temporarily, for instance in wounds, during regeneration of the endothelium, or during infection. When the endothelial barrier integrity is compromised, both the endothelium and the underlying tissues are more prone to infection by invasive pathogens, such as *S. aureus* [13–15]. Once internalized, *S. aureus* may persist or proliferate intracellularly for varying durations [16,17]. Intracellular persistence may lead to immune evasion and chronicity of infection, while intracellular replication of *S. aureus* will result in endothelial host cell lysis and spread into the underlying tissues [17, 18].

The human body is a key player in determining the outcome of infection, especially since host cell responses to close encounters with pathogens, such as *S. aureus*, differ per cell type. Accordingly, *S. aureus* has evolved mechanisms to manipulate the different host responses in order to survive in a wide range of hostile environments [4,19,20]. A variety of virulence factors and regulators allow *S. aureus* to breach cellular barriers and adapt to the intracellular environment. However, once the intracellular environment has been reached, mutual adaptations of the pathogen and its host will occur, as exemplified by the metabolic cross-talk that was observed upon the invasion of lung epithelial cells by *S. aureus* [18].

Understanding the behavior of different types of *S. aureus* in the intracellular compartments of different types of host cells is fundamental for developing adequate therapeutic approaches against chronic staphylococcal infections [21]. In this respect, our understanding of these processes is currently very limited. For instance, HA- and CA-MRSA can reach the blood stream via different routes, e.g. starting from infected surgical wounds or abscesses of the skin. To this end, closely related HA- and CA-strains of the *S. aureus* USA300 lineage have evolved different metabolic niche adaptations that favor their promulgation in blood or skin [10,22]. Yet, we do not know how such adaptations impact on the subsequent stages of the infection, where the bacteria enter and pass the endothelium. Therefore, the present study was specifically aimed at investigating the fate of internalized HA- and CA-strains of the USA300 lineage in endothelial cells. To this end, we established two *in vitro* infection models that take into consideration the condition of the endothelium at the moment of invasion and the subsequent course of infection. As a control for comparison, we included the well-defined laboratory strain *S. aureus* HG001 in our analyses [23]. The course of infection was followed quantitatively and qualitatively by flow cytometry as well as fluorescence and electron microscopy to determine eventual differences in the evolution of the infection over time among the different strains, as well as the final fate and intracellular distribution of the internalized bacteria.

## Materials and methods

### Biological materials

The *S. aureus* laboratory strain HG001 [24], and the clinical isolates D32 (CA-MRSA) and D53 (HA-MRSA) [10] were used to perform all experiments. The HG001 strain carried plasmid pJL-sar-GFP to express the green fluorescent protein [GFP; 25], and the D32 and D53 strains [10] carried plasmid pJL-sar-GFP_redopt-cm to express GFP. The culturing was performed in Roswell Park Memorial Institute 1640 medium (RPMI) (Gibco, New York), supplemented with 2 mM L-glutamine (Thermo Fisher Scientific, Waltham, USA). One day prior to infection, bacterial overnight cultures were prepared in serial dilutions in RPMI (Gibco, New York) supplemented with 0.01% yeast extract and 10 µg·ml^−1^ erythromycin or 10 µg·ml^−1^ chloramphenicol for the HG001 and USA300 strains respectively. The RPMI used for overnight precultures was supplemented with yeast extract to facilitate the initial growth of the bacteria, while antibiotics were added to prevent the possible loss of the GFP-encoding plasmids necessary for subsequent flow cytometry and fluorescence microscopy. Incubation was performed at 37°C and with constant shaking (250 rpm). The following day, exponentially growing overnight cultures were used to inoculate the main cultures for infection experiments using RPMI without yeast extract and antibiotics.

Primary human umbilical vein endothelial cells (HUVEC) from pooled donors (Lonza Cat# C2519a Lot# 394986, Allendale, NJ, USA) were used to perform all the experiments. The endothelial cells were grown in standard cell culture flasks (37°C, 5% CO_2_) in Endothelial Cell Growth Medium 2 [Ready-to-use; PromoCell, Germany). All experiments were performed using cells obtained from 3 to maximally 7 passages to avoid cell senescence and loss of primary cell characteristics. The medium was changed every 48 h.

### Internalization experiments and flow cytometry

Internalization experiments were performed as previously described by 26. Briefly, these experiments were performed using HUVEC seeded in 24-well plates (Greiner, Germany) pre-coated with rat-tail Collagen Type-I (Corning, New York). Two different conditions were applied, here referred to as “barrier” and “confluent”, following previously established protocols [27]. More in detail, to differentiate cells into a polarized endothelial cell barrier, HUVEC were seeded at a density of 3000 cells per cm^2^ and cultured for 7 days prior to infection, with media exchange every 2 days. For the confluent condition, HUVEC were seeded at a density of 50,000 cells per cm^2^ and cultured for 40 h prior to infection. The numbers of cells in the barrier and confluent culture conditions were counted prior to infection with *S. aureus* to verify that in both conditions 100,000 cells per well of a 24-well plate were infected.

A multiplicity of infection (MOI) of 25 was used for all internalization experiments. The bacterial master mix for infection was prepared from exponentially growing *S. aureus* cells in RPMI (OD_600_ of 0.4), which were counted by flow cytometry (see below), pelleted by centrifugation and resuspended in Endothelial Cell Growth Medium 2. The bacterial master mix was added to the HUVEC and the infected cells were incubated for 1 h (37°C, 5% CO_2_). Afterward, the medium was removed and replaced with Endothelial Cell Growth Medium 2 containing 25 µg·ml^−1^ of lysostaphin (AMBI Products, New York) to eliminate non-internalized bacteria bound to the HUVEC surface. The medium was changed every 48 h.

The abundance of human and bacterial cells was measured by flow cytometry. For this purpose, two groups of samples were collected in triplicate at different time points post infection (p.i.) up to 6 days. One sample group was used to quantify the number of host cells by treatment with trypsin-EDTA (Thermo Fisher Scientific, the Netherlands) during a 5 min incubation at 37°C, 5% CO_2_. Counting of infected HUVEC was performed with a Cytoflex S flow cytometer (Beckman Coulter, Woerden, the Netherlands) by excitation of GFP with a 488 nm laser and detection at 525/40 nm. Infected cells in the other sample group were lysed with 0.05% SDS for 5 min in order to collect intracellular bacteria. Analysis of the flow cytometry data was performed with Kaluza Analysis Software (Beckman Coulter, Woerden, the Netherlands). A gating strategy was applied to exclude debris and select healthy cells. Distributions and median-mean values of 20,000 cells were obtained. The relative number of infected HUVEC was expressed as the percentage of GFP-positive cells, and the number of intracellular bacteria was assessed by counting the number of GFP-positive events upon liberation of the intracellular bacteria from lysed HUVEC. Statistical analyses were performed with GraphPad Prism version 8 (GraphPad Software, La Jolla, CA, USA). Two-way Anova tests with multiple comparisons were performed to assess the statistical significance of differences in the numbers of internalized bacteria. P-values of ≤0.05 and a confidence of ≥95% were considered to indicate significance.

### Fluorescence microscopy

Immunofluorescence microscopy was performed using a Leica TCS SP8 Confocal laser scanning microscope (Leica Microsystems, Wetzlar, Germany). The cells were seeded over coverslips of 13 mm diameter #1.5 (Thermo Fisher Scientific, Waltham, USA). Coverslips with infected or uninfected cells were collected at different time points p.i. until 6 days and fixed with 4% formaldehyde for 20 min at room temperature. Subsequently, the cells were permeabilized, and blocked to avoid nonspecific antibody binding, by incubation for 20 min at room temperature with 0.5% Tween-20 in PBS, followed by overnight incubation at 4°C with 2% BSA and 5% neutral goat serum in PBS. Additional blocking was performed by incubation with 12 µg/ml of the human monoclonal antibody 1D9 [28], diluted in the same blocking solution, for 2 h at room temperature in a humidified chamber.

Subcellular localization of LAMP-1 was carried out by incubation with a primary mouse antibody against CD107a (LAMP-1; BD, United States) at a dilution of 1:100 for 1 h at room temperature in a humidified chamber. To detect the bound primary antibody, a secondary goat anti-mouse antibody conjugated with Alexa Fluor 594 (Invitrogen, Netherlands) was used at a 1:500 dilution with incubation for 1 h at room temperature. Lastly, DNA was stained with 4′,6-diamidino-2-phenylindole (DAPI; Roche, Switzerland). The slides were mounted with Mowiol 4–88 (Merk Millipore, USA) and stored at −20°C until microscopic visualization.

Tight junction proteins were immunostained to view their expression and distribution inside the cells upon growth under barrier or confluent conditions by confocal microscopy. The fixation, permeabilization and blocking procedures were carried out as described above. Subsequently, cells were incubated for 1 h at room temperature with a polyclonal rabbit primary antibody against the tight junction protein ZO-1 (zonula occludens-1, Life Technologies, NY, USA) at a dilution of 1:200 and a monoclonal mouse primary antibody against CD31 (PECAM-1; Dako, Glostrup, Denmark) at a dilution of 1:100. Bound antibodies were visualized by incubation for 1 h with a secondary goat-anti mouse antibody conjugated with Alexa Fluor 594 (Life technologies, NY, USA) at a 1:1000 dilution, or a donkey anti-rabbit antibody conjugated with Alexa Fluor 647 (Life technologies, NY, USA) at a 1:200 dilution. Image processing was performed using FIJI (https://fiji.sc/).

### Transmission electron microscopy

Cell samples for Transmission Electron Microscopy (TEM) were collected at 2 h, 7 h and 24 h p.i., and fixed with 0.2% glutaraldehyde and 2% paraformaldehyde in 0.1 M sodium cacodylate buffer (pH 7.4) for 1 h. The fixed cells were rinsed twice for 5 min in 0.1 M cacodylate buffer at room temperature followed by post-fixation in 1% osmium tetroxide, 1.5% potassium ferrocyanide in 0.1 M sodium cacodylate at 4°C for 30 min. The cells were then washed with Milli-Q water, dehydrated through serial incubation in a graded ethanol series (30%, 50%, 70% and 100%) and lastly embedded in EPON resin and polymerized at 37°C for 16 h followed by 58°C for 24 h. Ultrathin sections (80 nm) were cut with an UC7 ultramicrotome (Leica, Vienna, Austria) and contrasted using 5% uranyl acetate for 20 min, followed by Reynolds lead citrate for 2 min. Images were recorded with a CM100 Biotwin transmission electron microscope (FEI, Eindhoven, The Netherlands) operated at 80 kV using a Morada digital camera. Image processing was performed with FIJI (https://fiji.sc/).

### Apoptosis assay

To measure the induction of apoptosis in infected cells, activity of the apoptotic markers caspase-3 and −7 was measured at different time points p.i. (2 h, 7 h, 12 h, 24 h) using a commercial Caspase-Glo 3/7 assay kit (Promega, Madison, Wisconsin, United States) following the manufacturer’s instructions. Briefly, HUVEC were collected at different time points and 5000 cells were added to a white 96-well plate (Greiner, Germany) in duplicate. As a positive control, cells were treated with staurosporine (0.25 µM; Biaffin GmbH & Co KG, Germany) for 3 h. The caspase 3/7 substrates were added to each well and the plate was shaken at 300 rpm for 2 min followed by incubation in the dark for 30 min. Luminescence was quantified using a Synergy 2 multi-mode microplate reader (BioTek Instruments, Inc., Winooski, VT), and the results were expressed as n-fold induction relative to the uninfected control cells.

### Expression of staphylococcal panton-valentine leukocidin (PVL) receptors

To investigate the expression of PVL receptors in our HUVEC infection model, we used a flow cytometry assay based on staining with an allophycocyanin (APC)-labeled anti-human CD45 antibody and a PerCP/Cy5.5 anti-human CD88 (C5aR) antibody (BioLegend, United Sates). HUVEC were grown under barrier or confluent conditions as described above, and 1 × 10^6^ cells per sample were used for flow cytometry. The collected cells were incubated with the CD45- or CD88-specific antibodies according to the manufacturer’s predetermined optimum concentrations for 15–20 min at 4°C in the dark. Antibody incubation was followed by two washing steps with 2 ml of Cell Staining Buffer (BioLegend, United States), and centrifugation at 350 x *g* for 5 min. The cells were then fixed with 4% formaldehyde for 20 min at room temperature and resuspended in PBS. Flow cytometry measurements were performed with a Cytoflex S flow cytometer (Beckman Coulter, Woerden, the Netherlands). APC was excited with a 638 nm laser and fluorescence was recorded at 660/20 nm, whereas PerCP/Cy5.5 was excited with a 561 nm laser and fluorescence was recorded at 690/50 nm. Analysis of the flow cytometry data was performed with Kaluza Analysis Software.

Previous studies have shown that neutrophils express both the CD45 and CD88 (C5aR) receptors [29,30], and we therefore included neutrophils as a positive control for our flow cytometry experiments with HUVEC. Neutrophils were freshly isolated from healthy volunteers as previously described [31]. Briefly, the neutrophils were isolated from whole-blood samples using Lymphoprep buffer (StemCell Technologies, Vancouver, Canada) and gradient centrifugation to separate different cell types. After centrifugation, the plasma, Lymphoprep and peripheral blood mononuclear cells were removed, and a layer of erythrocytes and neutrophils was conserved. The erythrocytes were lysed with a red blood cell lysis buffer (10X; BioLegend, United states) followed by shaking for 10 min on ice and subsequent centrifugation. These two steps were repeated once to obtain a pellet of purified neutrophils. 1 × 10^6^ neutrophils were resuspended in 2 ml of RPMI 1640 medium (Gibco, Waltham, MA, USA) with 2 mM L-glutamine and 10% autologous donor serum and seeded in a 6- well plate. The neutrophils were allowed to rest on the plate at 37°C and 5% CO_2_ for 30 min. Subsequent flow cytometry experiments to detect CD45 and CD88, were performed with 1 × 10^6^ neutrophils per sample following the same procedure as described above for HUVEC. Additionally, we included the bronchial epithelial cell line 16HBE14o- in our PVL receptor expression studies. These cells have been used in previous studies to investigate the behavior of CA- and HA-MRSA isolates, or the control strain HG001, upon internalization [10, 18]. The lung epithelial cell line 16HBE14o- was cultured as described previously by [18]. Briefly, the cells were cultured at 37°C in 5% CO_2_ in eukaryotic minimal essential medium (eMEM; 1xMEM Biochrom AG, Germany) supplemented with 10% (v/v) fetal calf serum, 2 mM L-glutamine and 1% (v/v) non-essential amino acids 100x (Gibco, USA). For flow cytometry experiments 1 × 10^6^ cells per sample were used.

### Medical ethical approval

Blood donations from healthy volunteers were collected with approval of the medical ethical committee of the University Medical Center Groningen (approval no. Metc2012-375) after written informed consent and in accordance with the Helsinki Guidelines.

## Results

### S. aureus and the host endothelium

**Figure 1.**
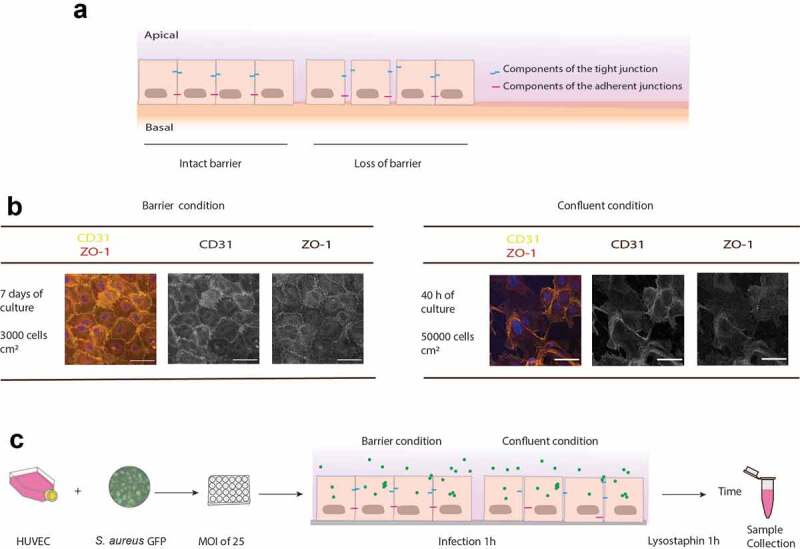
Schematic representation of the rationale and experimental setup to study *S. aureus* invading the endothelial barrier. (a) Endothelial cells are normally organized into a barrier with cell-cell junctions that control the passage of immune cells, molecules and pathogens. These cell-cell junctions include tight junctions, adherens junctions and a variety of adhesion molecules, such as ZO-1 and CD31 (PECAM-1). In a healthy condition, the barrier integrity is strictly maintained. However, endothelial cells may show loss of cell-cell junctions and this will lead to wound patches in the endothelial membrane. (b) Confocal fluorescence microscopy images of HUVEC in the barrier or confluent conditions stained with antibodies against ZO-1 (red in the merged image) and CD31 (yellow in the merged image). Blue: DAPI-stained nuclei. The micrographs present the maximum pixel value of the Z-stacks of the endothelial layers. Scale bar: 50 µm. (c) Experimental setup used during this study: two different endothelial conditions, referred to as “barrier” and “confluent”, were used for a 1-h infection with GFP-expressing *S. aureus*. Extracellular bacteria were removed by a 1-h incubation with lysostaphin. Subsequently, samples were collected at different intervals for time-resolved analysis of the infectious process

In the present study, we applied human primary umbilical vein endothelial cells (HUVEC) as a model for studying interactions of different *S. aureus* lineages with endothelial cell barriers at different states of integrity (schematically presented in Figure 1). Importantly, HUVEC can form tight junctions, polarize and differentiate into endothelial cell barriers [27,32,33]. To establish two endothelial barrier models at distinctive states of integrity, the cells were cultured on a collagen I matrix to different cell densities and for different time periods. As previously shown by Francia et al, HUVEC can be differentiated into a cell barrier by seeding cells at a low density (3000 per cm^2^) and culturing for 7 days [27]. In contrast, HUVEC in a confluent condition can be obtained by seeding at a higher cell density (50,000 per cm^2^) and culturing for only 40 h. The formation of monolayers and tight junctions was monitored using two common tight junction proteins, namely ZO-1 and CD31. At 7 days post seeding, HUVEC in the barrier condition showed clear tight junctions at the contact sites of the cells (Figure 1(c)), reflecting the formation of an endothelial cell barrier, as previously demonstrated [27]. In contrast, in the confluent condition, at 40 h post seeding, the tight junction proteins were detectable all over the cells and a completely organized monolayer was absent (Figure 1(c)). Thus, this confluent condition was used as a model for compromised endothelial barriers, as can be the case for blood vessels damaged by surgery or trauma. We emphasize that the final cell number per well in both cases was the same. However, because of the different conditions, only in the 7-day cultures had the cells formed a polarized cell barrier [27].

To investigate the fate of *S. aureus* internalized in the two endothelial cell models, we selected three different strains, namely the CA-MRSA strain D32, the HA-MRSA strain D53, and the laboratory strain HG001, all of which expressed GFP (Figure 1). The two MRSA strains are clinical isolates belonging to the USA300 lineage, which were recently characterized in-depth by comparative genome analyses and proteomics [10,22]. As shown previously, these two strains with different epidemiology display metabolic adaptations that have an impact on virulence factor expression and survival inside lung epithelial cells. The HG001 control strain is a derivative of *S. aureus* NCTC8325, isolated from a patient with sepsis. In *S. aureus* HG001 the *rsbU* gene, encoding a positive activator of sigma B, has been repaired to allow its usage as a versatile model for studies on gene regulation and pathogenicity [24,34].

The experimental setup used to assess the fate of the three selected *S. aureus* strains upon attachment and internalization by HUVEC in the barrier or confluent states is schematically represented in Figure 1(c). Of note, studies in the barrier model were limited in time due to the loss of a clear tight-junction organization at the interface of the cells after 48 h, also in the absence of infecting bacteria. Thus, the bacterial fate could be followed for up to 48 h p.i. in the barrier model, and even up to 144 h p.i. in the confluent model. Furthermore, the bacteria used for the infection experiments were precultured in RPMI medium, in order to tune their physiological state toward a bacteremia condition. The RPMI medium was selected based on the results from a previous study, where *S. aureus* HG001 cultured under many different conditions was analyzed by transcript profiling, showing that the gene expression signatures of the bacteria grown in human plasma or RPMI were highly similar [23].

### The differentiation into endothelial cell barriers determines rates of infection and bacterial survival

**Figure 2.**
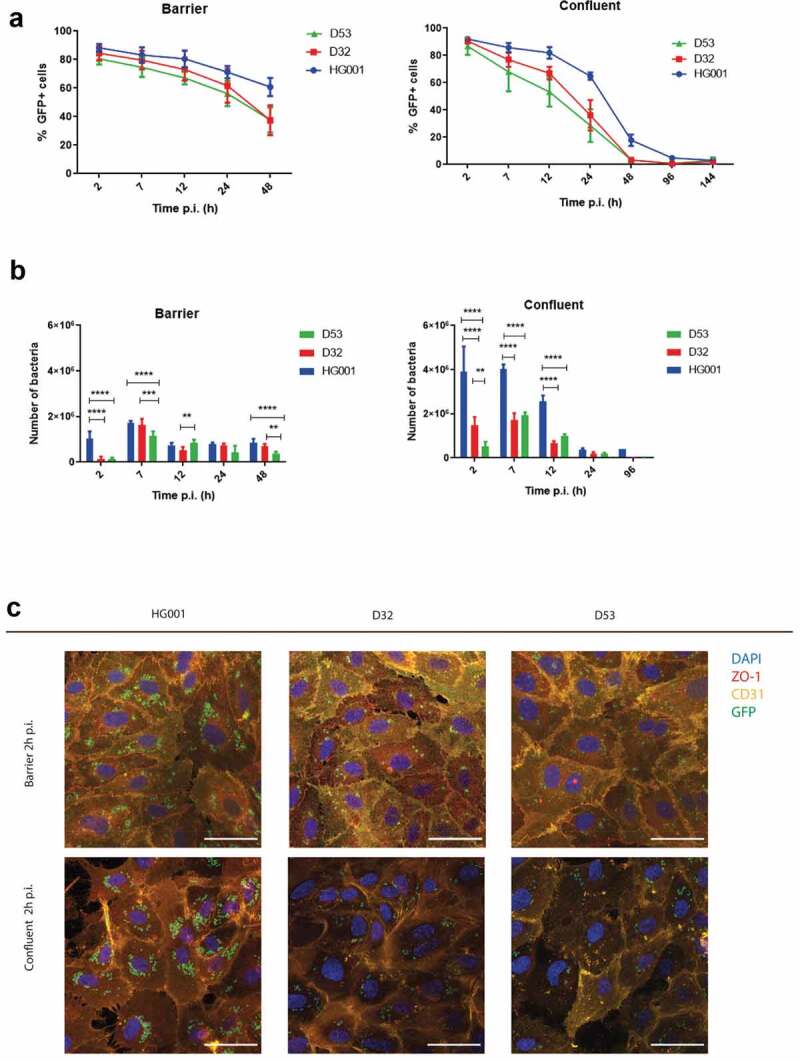
The presence of intact junctions between endothelial cells determines rates of infection and bacterial survival. The progression of infection in the barrier and confluent conditions by GFP-expressing bacteria of *S. aureus* strains HG001, D32 or D53 was followed by flow cytometry (a and b) and fluorescence microscopy (c). (a) The % of GFP-positive host cells in the total cell population was determined over 48 h for the barrier and 144 h for the confluent condition. (b) The numbers of internalized bacteria per well of a 24-well plate with infected cells were counted over time. Two-way Anova with multiple comparisons was performed to assess the significance of differences in the bacterial numbers for different strains at particular time points of sampling. * (p < 0.03), ** (p < 0.002), *** (p < 0.0002), **** (p < 0.0001). (c) Confocal fluorescence microscopy images of HUVEC cells in barrier and confluent conditions infected with *S. aureus* (green). The HUVEC were stained at 2 h p.i. with anti-ZO1 (red in merged the image) and anti-CD31 (yellow in merged the image) antibodies. Blue: DAPI-stained nuclei. The micrographs present the maximum pixel value of the Z-stacks of the endothelial layers. Scale bar: 50 µm. For the unmerged images and controls with uninfected cells, see Supplemental Figure S2

HUVEC that had reached the barrier or confluent states were infected for 1 h with *S. aureus* and, subsequently, any non-internalized bacteria were killed by the addition of lysostaphin. The course of infection was then followed by flow cytometry to quantify both the host cell population and the population of internalized bacteria over time (Figure 2, Supplemental Figure S1). Of note, the presence of lysostaphin in the medium prevents bacterial survival outside cells, thus impairing the re-infection of nearby host cells.

For the barrier infection model, samples were collected at 2, 7, 12, 24 and 48 h p.i. During the first 24 h of infection, the infected host cell population and the internalized bacteria showed important changes (Figure 2(a,b)). While the percentage of GFP-positive infected host cells dropped from 90% to about 60% for the HG001 and from 80% to 40% for the USA300 strains of the total gated single cell population, the internalized bacterial population started to grow especially during the first 7 h p.i. and remained relatively stable thereafter at a slightly lowered level. Here it should be noticed that, in the first 2 h p.i., the number of internalized bacteria was statistically significantly higher for the HG001 strain compared to the CA- and HA-MRSA strains D32 and D53, respectively. However, by 7 h p.i. the intracellular D32 population had reached comparable numbers as the HG001 population, while the D53 population remained somewhat smaller in numbers. Consistent with the higher numbers of internalized HG001 bacteria, higher numbers of GFP-positive HUVEC were detected over time upon infection with this strain, particularly at 48 h p.i. (Figure 2(a,b)).

Compared to the barrier infection model, a very different infection dynamic was observed in the confluent infection model. In the first place, the number of GFP-positive cells declined more rapidly and, at 48 h p.i., merely 20% of the cell population was GFP-positive when infected with the HG001 strain compared to over 90% observed at 2 h p.i. (Figure 2(a)). At this same time, the numbers of GFP-positive HUVEC infected with the D32 or D53 strains were even close to zero (Figure 2(a)). Additionally, as observed in the case of the barrier model, also in the confluent infection model, the HG001 strain reached the highest numbers of internalized bacteria, right from 2 h p.i. up until 96 h p.i. Further, at 2 h p.i., higher numbers of internalized bacteria were observed for the D32 strain compared to the D53 strain, but this difference was no longer detectable at later time points p.i. (Figure 2(b)). These time-dependent changes in the internalized bacterial populations were mirrored in the numbers of GFP-positive HUVEC (Figure 2(a)).

Important differences in the infection progression were observed when comparing the confluent and barrier infection models. In particular, the number of internalized bacteria of the HG001 strain in the confluent model was nearly three-fold higher than in the barrier model at 2 h p.i., and it remained higher during the first 12 h p.i. (Figure 2(a,b)). A similar trend was observed for the D32 and D53 strains, albeit that in both models the numbers of intracellular bacteria at 2 h p.i. were much lower than the respective numbers of the HG001 strain. Conversely, the intracellular numbers of the D32 and D53 strains were higher in the barrier infection model than in the confluent model from 24 h p.i. onwards. However, at 7 and 12 h p.i., the numbers of internalized bacteria of the D32 and D53 strains in the two infection models were more similar. Another remarkable difference for the barrier and confluent HUVEC cell infection models was the fact that, compared to 7 h p.i., relatively fewer bacteria survived internalization in the confluent infection model from 24 h p.i. onwards, in comparison to what was observed in the barrier at the same time points. In accordance with this decline in bacterial viability, the numbers of GFP-positive cells started to decline significantly already at 12 h p.i. This implies that in the confluent model, the bacteria are eliminated either by the HUVEC themselves intracellularly, or by “suicidal escape” from the HUVEC and subsequent extracellular killing by lysostaphin, or both. Altogether, these results suggest that the different states of HUVEC when grown to confluence, or differentiated into an endothelial cell barrier, determine the rate of bacterial internalization, the numbers of internalized bacteria, the percentage of infected cells, and the long-term survival of the bacteria inside the HUVEC. Indeed, the differentiation of HUVEC into a cell barrier leads to the formation of cell-cell junctions, which is a dominant phenotypic feature of cell barriers. However, this differentiation is accompanied by other changes that can also affect the infection and the course of its evolution. For example, endocytosis markers are expressed differently by cells in the barrier and confluent states [27]. Of note, irrespective of the investigated strain, the long-term survival of internalized bacteria was much higher in the barrier infection model than in the confluent model. This implies that an intact barrier has a higher propensity to sustain persistent intracellular infection. Yet, in both models, the HG001 strain infected the highest numbers of HUVEC and it displayed the highest intracellular persistence, showing that there are clear strain-specific differences in intracellular survival. This was also clearly observed by confocal fluorescence microscopy, where the HUVEC grown to confluence or differentiated into a barrier were immuno-stained at 2 h p.i. to visualize ZO-1 and CD31 (Figure 2(c), Supplemental Figure S2). Of note, even in the barriers formed by the HUVEC, some “gaps” and concomitant loss of tight cell-cell contacts were detectable after bacterial infection (Figure 2(c) and S2). Such gaps were close to absent from barriers formed by uninfected control cells (Figure 2(c) and S2), which implies that infection did compromise the integrity of the HUVEC barrier to some extent. However, no significant differences were detectable for the three strains, indicating that they have a comparable ability to break this barrier.

### *HUVEC-internalized* S. aureus *resides in LAMP-1-positive membrane-enclosed compartments*

**Figure 3.**
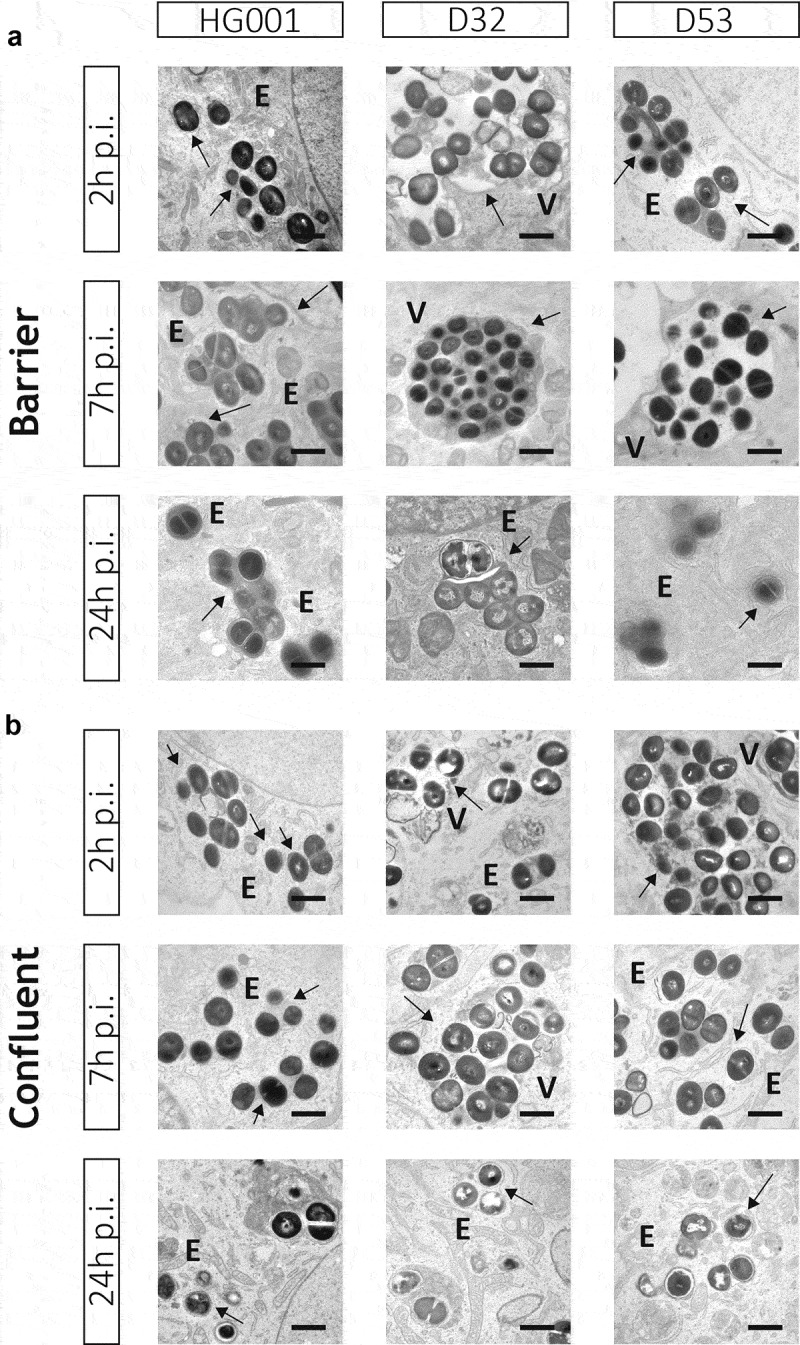
Bacteria internalized by endothelial cells remain localized within membrane-enclosed compartments. Transmission electron microscopy images of HUVEC infected with the *S. aureus* strains HG001, D32 or D53 in the barrier (a) and confluent (b) conditions. In both conditions, the investigated *S. aureus* strains show replication from 2 h to 7 h p.i. Electron-dense (e) compartments of different sizes and vacuole-like structures (v) are indicated in the different panels. No cytoplasmic bacteria were detectable in both conditions. Arrows indicate replicating bacteria. Scale bar : 1 µm. For additional images, see Supplemental Figure S3

Previous studies have shown that *S. aureus* can reside both in the cytoplasm and various other subcellular compartments of human host cells [20,35–39]. To obtain a deeper understanding of the destiny of *S. aureus* inside HUVEC in the barrier and confluent infection models, we used TEM (Figure 3, Supplemental Figure S3). Interestingly, the TEM analyses showed that the bacteria resided exclusively in membrane-confined compartments. These included both large electron-lucent vacuole-like structures and more electron-dense compartments of variable size. The larger electron-dense compartments contained big clusters of bacteria, whereas the smaller electron-dense compartments contained only one or few bacteria. The bacteria present in big clusters frequently showed division planes, suggesting that they were replicating inside the enclosed compartments. Judging by previous studies, the electron-dense compartments are possibly lysosomes or phagolysosomes, where the main degradation processes of the host cells are taking place [40, 18]. Since no cytosolic (i.e. “membrane-free”) bacteria were observed, it seems that upon invasion of the HUVEC, the investigated *S. aureus* strains D32, D53 and HG001 preferentially adapt to lysosome- or vacuole-like organelles of the host-cells, rather than to “escape” to the cytosol. However, in this respect it should be mentioned that, conceivably, different preculturing conditions of the bacteria might tune them for a more aggressive invasive state. For example, previous endothelial cell infection experiments with *S. aureus* strains precultured in tryptic soy broth (TSB) or Muller Hinton (MH) broth showed phagolysosomal escape [20,35–39]. This could possibly relate to higher levels of virulence factor production in TSB or MH broth compared to the presently used RPMI medium, but it might also relate to the use of different cell types for the infection experiments.

Interestingly, from 2 to 7 h p.i., when the replication of internalized bacteria was highest, bacteria of the D32 and D53 strains were mostly found in clusters within large vacuolar structures. At later times, these bacteria were detected mostly in smaller numbers within lysosome-like organelles. On the contrary, bacteria of the HG001 strain were detected mostly in small numbers within small electron-dense lysosome-like organelles, right from 2 h p.i. Importantly, this subcellular localization, i.e. inside lysosome- or vacuole-like compartments, was observed both for the barrier and confluent infection models (Figure 3, Supplemental Figure S3).

**Figure 4.**
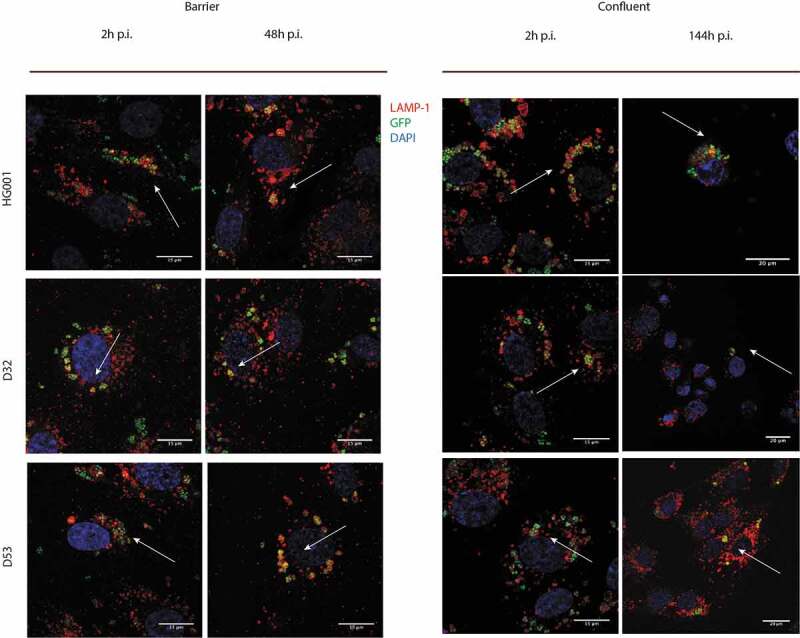
*S. aureus* internalized by endothelial cells co-localize with the lysosomal-associated membrane protein 1 (LAMP-1). Confocal fluorescence microscopy images show HUVEC infected with the *S. aureus* strains HG001, D32 or D53 in the barrier (a) and confluent (b) conditions stained with an anti-LAMP-1 (red) antibody. Blue: DAPI-stained nuclei. Green: GFP-expressing bacteria. Co-localization with LAMP-1 was observed from the beginning of infection (2 h) until the end of the observation time (48 h for the barrier condition and 144 h for the confluent condition) for all three strains. Arrows indicate the co-localization. For enlarged images, see Supplemental Figure S4, and for images recorded at different time points p.i. see Supplemental Figure S5

To further study the intracellular distribution of *S. aureus*, we performed confocal fluorescence microscopy to determine eventual co-localization with LAMP-1, a protein recruited to both lysosomal, phagolysosomal and vacuolar membranes [36,41,42]. Indeed, the GFP-expressing bacteria co-localized with LAMP-1-positive compartments over the entire time period of observation (i.e. from 2 h to 144 h p.i.), both in the barrier and confluent infection models, and irrespective of the investigated strain (Figure 4; Supplemental Figures S4 and S5). We therefore conclude that this subcellular localization is typical for both investigated HUVEC models, and that it is thus not influenced by the differentiation of cells into a polarized endothelial cell barrier.

### *Activation of caspase 3/7 during intracellular* S. aureus *replication*

**Figure 5.**
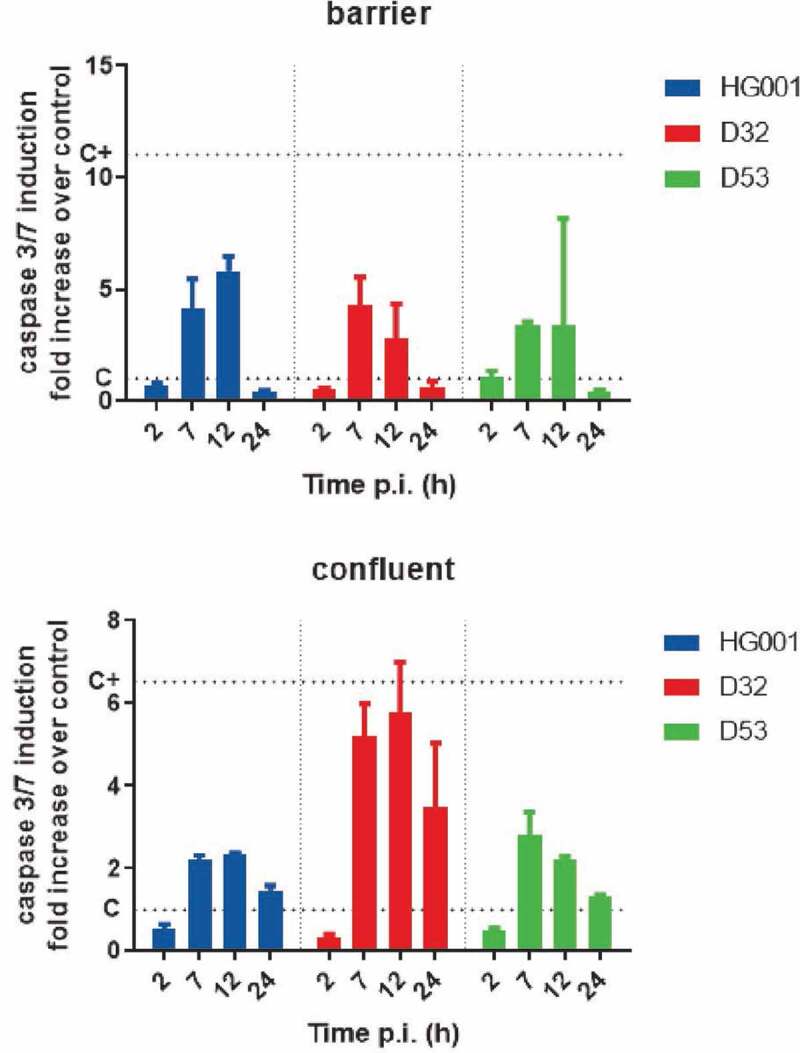
Infection of endothelial cells with *S. aureus* induces apoptosis. The induction of apoptosis in HUVEC infected with the *S. aureus* strains HG001, D32 or D53 in the barrier or confluent conditions was inspected by measuring the activity of the apoptotic markers caspases 3 and −7 at different time points p.i. The graphs show the caspase 3/7 induction fold increase over the control with uninfected cells. For additional controls, see Supplemental Figure S6

The experiments described above show that HUVEC in the barrier model maintained their viability for at least 48 h, and HUVEC in the confluent model for at least 144 h. Also, upon infection with the *S. aureus* strains D32, D53 or HG001, the integrity of host cells with internalized bacteria was not detectably affected, although the HUVEC barrier did display some gaps (Figure 2, Supplemental Figure S2). Furthermore, the internalized *S. aureus* multiplied during the first 7 h p.i., but thereafter the bacterial numbers declined. From 12 h p.i., the bacterial numbers in the barrier model remained largely constant, whereas they were reduced to near-zero in the confluent model. Together, these observations suggested that the bacteria, while initially replicating in the lysosome- or vacuole-like compartments, were eventually eliminated in the confluent model or forced into a non-replicating state in the barrier model. Yet, it was conceivable that, due to apoptosis, part of the bacterial population was liberated from the HUVEC, leading to their destruction by the lysostaphin that had been added to the cell culture medium to prevent reinfection by escaping bacteria. To check whether, as part of the intrinsic immune defense mechanism of the HUVEC, some cell death signaling pathways were activated by bacterial effectors and toxins, we employed the Caspase 3/7 assay. This assay allows the visualization of apoptotic events based on the bioluminescent detection of caspase activity. Of note, apoptosis represents a non-inflammatory type of cell death that can be triggered by intrinsic (mitochondria-mediated) or extrinsic (receptor-mediated) pathways, but these two pathways converge in the activation of caspases 3/7. Further, it is important to bear in mind that the caspases 3/7 are the major executioner caspases that initiate the hallmarks of the degradation phase of apoptosis, especially DNA fragmentation, cell shrinkage and membrane blebbing. Using the caspase 3/7 assay, we observed, for all strains and in both endothelial models, some caspase activation between 7 and 12 h p.i. (Figure 5), which corresponds with the replicating phase of the internalized bacteria (Figure 2). The highest degree of caspase 3/7 activation was observed for the D32 strain in the confluent infection model, whereas the three investigated strains caused comparable levels of caspase activation in the barrier model. Caspase activation decreased at 24 h p.i., when the numbers of internalized bacteria also decreased (Figures 2 and 5). Of note, we also verified whether the Caspases 3/7 signal that we observed was exclusively due to cleavage of the proluminescent caspase-3/7 DEVD-aminoluciferin substrate by HUVEC-specific caspases. Indeed, as shown in Supplemental Figure S6, the infecting *S. aureus* cells did not produce proteolytic activities that could interfere with the assay outcome by cleavage of the DEVD-aminoluciferin substrate. Together, these observations imply that, in particular, the replicating bacteria trigger some apoptotic events, but that apoptosis cannot be the major reason why the population of internalized bacteria decreases at late time points of infection for all strains and in both endothelial models used. From this we infer that the bacteria are eliminated inside the lysosome- or vacuolar-like compartments unless they reach a non-replicating state in the barrier model.

### Expression of the staphylococcal PVL receptors in HUVEC

One of the hallmarks of CA-MRSA isolates is the production of the leukotoxin PVL [43]. In contrast, the PVL-encoding *lukFS* genes are generally absent from HA-MRSA isolates. In agreement with this, we have previously shown that the CA-isolate D32 used for this study produces PVL, whereas the HA-isolate D53 lacks the respective *lukFS* genes [10]. Since only relatively minor differences were observed in the infection of HUVEC by the D32 and D53 strains, we wondered whether these cells would express the PVL receptors CD88 [C5aR; 29] and CD45 [30]. In particular, binding of the S-component of PVL to CD88 and CD45 was previously shown to contribute to the cellular tropism and human specificity of this toxin [29,30]. This can result in pore formation in the eukaryotic cell membranes and host cell lysis.

**Figure 6.**
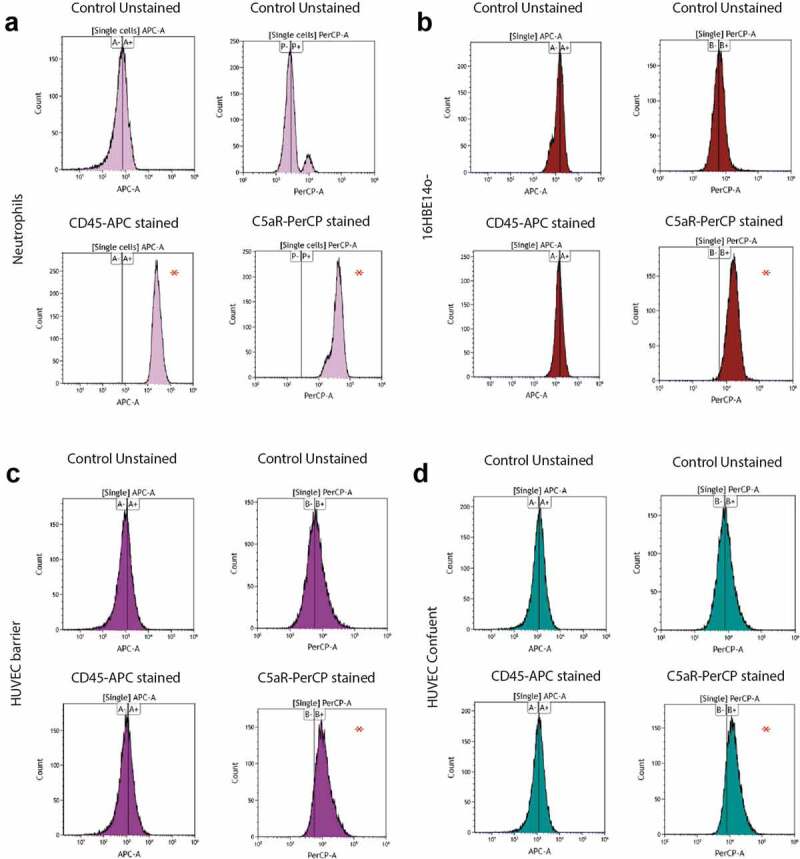
PVL receptor expression in neutrophils, lung epithelial cells and HUVEC. To investigate the expression of receptors for the secreted staphylococcal toxin PVL, a flow cytometry-based cell staining assay was applied using APC-labeled anti-human CD45 and PerCP/Cy5.5-labeled anti-human CD88 (C5aR) antibodies. (a) Human neutrophils (positive control), (b) bronchial epithelial cell line 16HBE14o-, (c) HUVEC in the barrier condition, and (d) HUVEC in the confluent condition. HUVEC and bronchial epithelial cells tested negative for CD45-staining, but showed shifts in fluorescence intensity upon CD88 (C5aR)-staining. Neutrophils showed major shifts in fluorescence intensity upon staining for CD45 and CD88. * indicates the observed shifts in fluorescence intensity upon staining with the anti-CD45 and anti-CD88 antibodies

To measure expression of the CD88 and CD45 receptors in our endothelial model systems, we used a flow cytometry assay based on an APC-labeled anti-human CD45 antibody and a PerCP/Cy5.5-labeled anti-human CD88 antibody (Figure 6). In addition, we used human neutrophils as a positive control for receptor expression and 16HBE14o- lung epithelial cells that we employed in our previous studies [44, 18] for comparison with the HUVEC. Indeed, as previously reported [29,30], clear CD88 and CD45 signals were observed in neutrophils (Figure 6(a)). In contrast, the HUVEC in the barrier and confluent conditions did not show a detectable fluorescence intensity shift compared to the unstained sample for the CD45 antibody, and only a minor fluorescence intensity shift for the CD88 antibody (Figure 6(c,d)). These observations imply that the CD45 receptor is absent from HUVEC and that CD88 is present only in relatively small amounts compared to neutrophils. Similarly, CD45 was absent from the 16HBE14o- lung epithelial cells. CD88 was however detected in the lung epithelial cells at levels that were clearly higher than those in HUVEC, but at lower levels than the CD88 detected in neutrophils (Figure 6(b)). Importantly, the observed low-level expression of the CD88 PVL receptor in HUVEC could explain the relatively small overall differences observed in the infection of HUVEC by the CA-MRSA isolate D32 and the HA-MRSA isolate D53. In addition, the higher expression of CD88 in the 16HBE14o- lung epithelial cells would be consistent with the differential behavior of these CA- and HA-MRSA isolates in the latter infection model [10].

## Discussion

The present study was aimed at analyzing the impact of *S. aureus* internalization on human endothelial cells and the subsequent fate of the internalized bacteria. Our results show that the bacteria are readily internalized, but get trapped in membrane-enclosed compartments where they either fade away or reach a low/non-replicating state. This is in stark contrast with what we previously observed for 16HBE14o- lung epithelial cells, where internalized bacteria managed to escape to the cytoplasm and then either lysed the host or became physiologically “dormant” [18]. It thus seems that, at least in the present experimental setup, the employed endothelial cells (i.e. HUVEC) are much better able to contain the invading *S. aureus* bacteria than 16HBE14o- lung epithelial cells. Whether the non-replicating *S. aureus* bacteria inside the membrane-enclosed compartments of the HUVEC have reached a genuine state of dormancy with an altered metabolic profile, as previously shown for *S. aureus* that have escaped to the cytoplasm of lung epithelial cells, remains to be investigated in more detail.

The dynamics of *S. aureus* intracellular infection in the two presently implemented endothelial models reflects well the *in vivo* course of infection. As shown by flow cytometry, the number of internalized bacteria in the confluent HUVEC infection model was higher than in the barrier model at 2 h p.i., and this difference was maintained up to 12 h p.i. One possible explanation of this difference relates to the fact that bacteria infecting HUVEC differentiated into a cell barrier, first have to pass the cell-cell junctions before they can reach integrins exposed on the HUVEC. Of note integrins are usually exposed on the basolateral side of endothelial cells and their binding is necessary for staphylococcal invasion [45]. In fact, many bacteria produce toxins, which can kill endothelial cells, weakening their cytoskeleton and opening the cell-cell junctions [46]. In contrast, when the HUVEC are merely confluent, the tight junction proteins are intracellular and do not form a clearly structured organization, which will give the bacteria faster access to the host cells and their intracellular environment. This has implications for the situation in the human body, where the endothelium normally controls permeability. If the organized structure of the endothelium is lost, which can happen upon trauma, the distribution of integrins and junction proteins that are usually present at the basolateral side of polarized endothelial barriers is lost; thus, they can be exposed on all sides allowing bacteria to bind and invade them at higher levels [47,48]. Additionally, as shown by 27, polarized HUVEC in a barrier display lower mRNA expression levels of endocytic targets and lower nanoparticle uptake compared to cells in the confluent condition. An intact barrier is, thus, not only fundamental for protection against bacterial infection, but also sets limits to the uptake of much smaller objects, such as nano-sized carriers.

Once *S. aureus* had reached the inside of the endothelial cells, be it under the barrier or confluent conditions, it was retained in membrane-enclosed compartments resembling vacuoles, phagolysosomes and lysosomes. Importantly, immunofluorescence microscopy revealed that the GFP-positive *S. aureus* compartments were also LAMP-1-positive, and colocalization of the bacteria with LAMP-1 was maintained during the entire period of observation in both the barrier (2 days p.i.) and confluent states of the endothelial cells (6 days p.i.). This was observed for all three investigated *S. aureus* strains, which implies that, once the bacteria become internalized in endothelial cells, regardless of barrier formation, they remain confined in LAMP-1-positive compartments. Thus, it seems that those staphylococci that survive inside the HUVEC for extended periods of time manage to adapt to the degradative compartments of their host cells, whereas they do not establish themselves in the cytoplasm. At present, we cannot say whether the internalized bacteria do not reach the cytoplasm at all, or whether some do reach the cytoplasm and then get killed in this compartment, which is generally considered a less extreme environment than the interior of (phago)lysosomes or the vacuole. In fact, bacterial replication was observed inside these membrane-enclosed intracellular compartments from 2 h to 7 h p.i.

Despite the general similarities that we observed upon infection of HUVEC by the three investigated *S. aureus* strains, we observed also clear strain-specific differences in terms of internalization rate, the percentage of infected cells, and the long-term survival of the bacteria inside the HUVEC. In particular, barriers with polarized cells and cell-cell junctions were more resistant to infection than the confluent HUVEC but, once internalized, the bacteria had a higher propensity to reach a state of persistence in the barrier condition. In contrast, for compromised endothelial cell barriers, we observed much higher rates of bacterial internalization, which was potentially more toxic for the cells. This would explain why the numbers of internalized *S. aureus* dropped almost to zero over time in the confluent infection model. Lastly, although the numbers of bacteria that entered the HUVEC were smaller for the D32 and D53 strains compared to strain HG001, they reproduced much faster during the first 7 h p.i., especially in HUVEC that had formed a barrier. This shows that even the internalization of very few bacteria may turn into a rather persistent intracellular infection, and this effect was actually most pronounced for the clinical D32 and D53 strains.

Our present observations are in accordance with previous studies in different cell types, where intracellular replication of *S. aureus* in organelles, such as vacuoles, phagosomes, phagolysosomes, and autophagosomes was observed [20,37,49,50]. In our present experimental setup, from 24 h p.i. onwards, the internalized bacteria seemed to stop replicating and relatively low numbers of bacteria persisted until 6 days p.i. Intracellular persistence has been described for several species of bacteria and cell types, where the bacteria are able to remain viable in the host for prolonged periods of time [51–53]. Survival of *S. aureus* in endothelial cells has been observed until 10 days p.i [16]. At present, we do not know what determines the duration of intracellular survival of *S. aureus* in terms of strain- and host cell type-specific differences. However, at the bacterial end, we can envisage that stress management, metabolic adaptations, and cytolytic toxin production are prime parameters. For instance, the HG001 strain included in our present studies was “modified” such that all possible defects in gene regulators, particularly *rsbU* related to the SigB response, were repaired [24]. This may explain why this strain showed a superior capability to invade the HUVEC and to survive intracellularly compared to the two investigated clinical HA- and CA-USA300 isolates, which were previously shown to display differential expression of the respective SigB regulons [22]. In addition, it was previously shown that another *S. aureus* RsbU^+^-repaired strain (SH1000) also displayed increased internalization and intracellular growth [54].

For the two investigated USA300 strains, we recently identified niche-specific metabolic adaptations priming the HA-strains for growth in nutrient-proficient environments, whereas the CA-strains were more geared toward growth in nutrient-deplete environments [22]. Such adaptations may provide also advantages for intracellular growth and survival, because the internalized bacteria need to compete with their host for nutrients, especially when they reside intracellularly for extended periods of time. This may explain why the CA-isolate D32 managed to establish intracellular growth in HUVEC somewhat faster than the HA-isolate D53, especially at early times p.i. On the other hand, we previously showed clear differences in the expression of cytolytic toxins by the two strains, especially for phenol-soluble modulins (PSMs), which were produced to much higher levels by the HA-isolate D53 than the CA-isolate D32 [10]. Conversely, the CA-isolate D32 expresses the Panton Valentin Leukocidin (PVL), whereas the HA-isolate D53 lacks the *lukFS* genes for PVL [10]. Such differences in toxin production may also impact on intracellular growth of *S. aureus*, since they could for instance enhance host cell lysis, leading to the suicidal escape of the bacteria into an extracellular environment that was supplemented with lysostaphin. Yet, the difference in PVL production by the D32 and D53 strains is probably of minor overall importance in the infection of HUVEC as the PVL receptors CD45 and CD88 were, respectively, absent or present only in low amounts in these cells. Moreover, judging by the assessment of caspase 3/7 activation, the internalized bacteria did not contribute in major ways to caspase activity-related cell death, except perhaps in the confluent cells, where the caspase 3/7 activation by the PVL-proficient CA-strain D32 was higher than the activation of these enzymes by the PVL-negative strains D53 and HG001. Yet, this difference was not detected in HUVEC that had formed a barrier. Taken together, it seems however that effective stress management and metabolic adaptations are more important features for intracellular growth and survival in HUVEC than the production of cytolytic toxins.

## Conclusion

Altogether, our present observations provide a better insight into how different *S. aureus* strains can take advantage of endothelial cells to survive intracellularly for a prolonged period of time. Our study in fact highlights the different dynamics of *S. aureus* infection in two endothelial models, which mimic two different conditions of the endothelium. We further conclude that the dynamics and localization of intracellular *S. aureus* shows important strain- and host cell type-specific differences. Importantly, compared to our previous infection studies with the same staphylococcal strains in lung epithelial cells, we notice clear differences in the subcellular localization of the internalized bacteria and the duration of their intracellular survival. While the bacteria remained enclosed in (phago)lysosomes or vacuole-like compartments of HUVEC, they managed to escape from these compartments in the lung epithelial cells [18]. In addition, we previously observed much stronger differences in the internalization dynamics of the CA- and HA-isolates in lung epithelial cells than in the here investigated HUVEC cells [10], which may well relate to particular metabolic adaptations [22], as well as differences in the levels of the PVL receptor CD88 between HUVEC and the lung epithelial cells. Finally, a major conclusion that can be drawn from our present study is that the low-level invasion by *S. aureus* of endothelial cells in a tightly sealed barrier state is more likely to lead to persistent staphylococcal infection than invasion of a damaged endothelium by higher numbers of bacteria. This would be consistent with a clinical scenario where disruption of the endothelium by trauma may lead to severe invasive *S. aureus* infection of the underlying tissues, while chronic infections by *S. aureus* are generally associated with low bacterial counts and less fulminant pathology.

## Supplementary Material

Supplemental Material
