# Supplementary material for: Time-resolved analysis of Staphylococcus aureus invading the endothelial barrier
Source: Virulence. 2020 Nov 22;11(1):1623–39. doi: 10.1080/21505594.2020.1844418 (PMC7714425; doi:10.1080/21505594.2020.1844418)
Supplement: Supplemental Material [file KVIR_A_1844418_SM7396.pdf]

Supplemental Figures

Time-resolved analysis of *Staphylococcus aureus* invading the endothelial barrier

Elisa J.M. Raineri[1#], Harita Yedavally[2#], Anna Salvati[2*] Jan Maarten van Dijl[1*]

[1]Department of Medical Microbiology, University of Groningen, University Medical Center Groningen, Hanzeplein 1, 9700 RB, Groningen, The Netherlands
[2]Department of Nanomedicine and Drug Targeting, Groningen Research Institute of Pharmacy, University of Groningen, A. Deusinglaan 1, 9713 AV, Groningen, The Netherlands

# These authors contributed equally to the work.

***Corresponding authors:**
Prof. Dr. Jan Maarten van Dijl, Dept. of Medical Microbiology, University of Groningen, University Medical Center Groningen, P.O. Box 30001, 9700 RB Groningen, the Netherlands, Tel. +31-50-3615187, E-mail j.m.van.dijl01@umcg.nl.
Prof. Dr. Anna Salvati, Department of Nanomedicine and Drug Targeting, Groningen Research Institute of Pharmacy, University of Groningen, A. Deusinglaan 1, 9713 AV, Groningen, The Netherlands, Tel. +31-50-3639831, E-mail a.salvati@rug.nl

Running title: Kinetics of *S. aureus* endothelial cell invasion

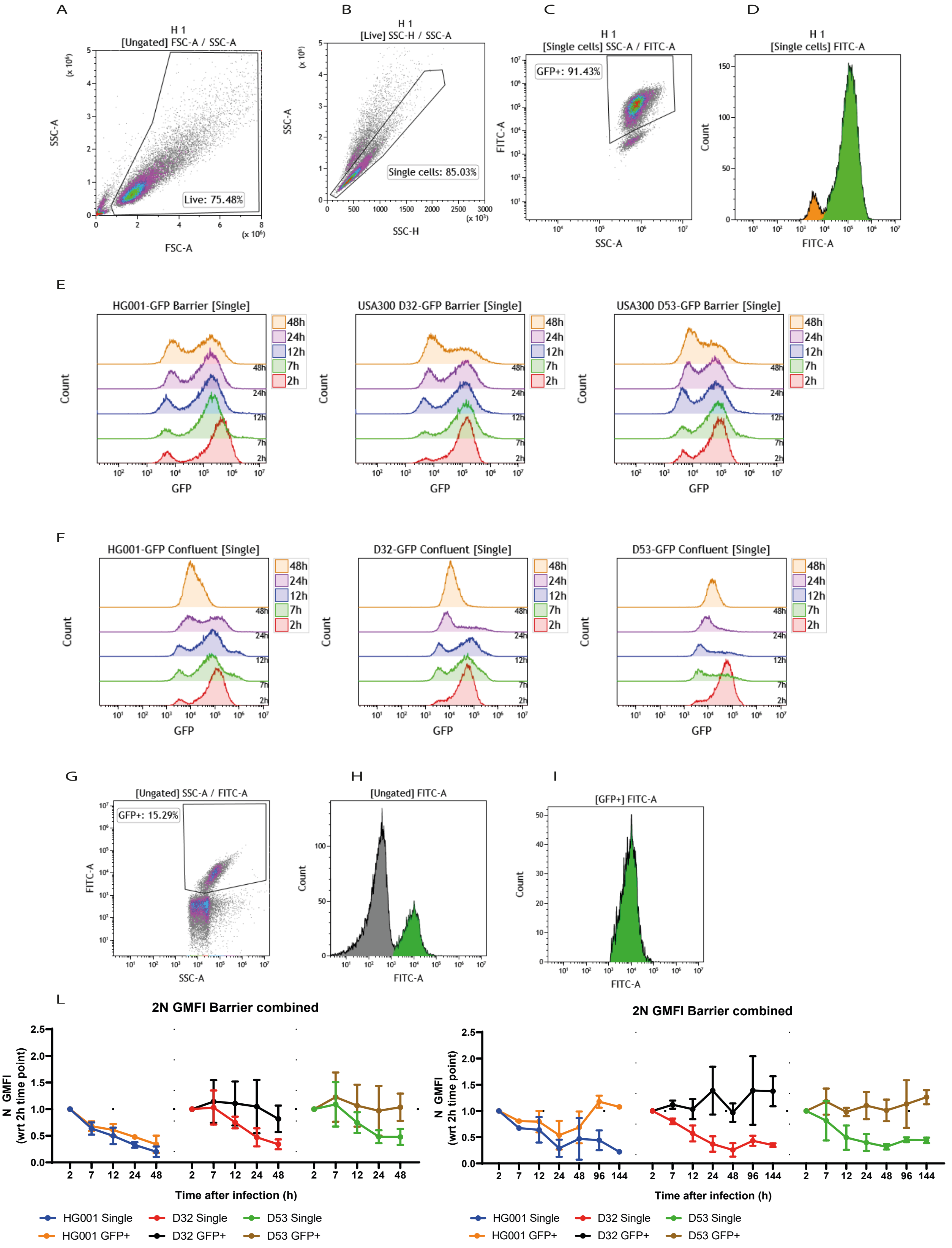

Supplemental Figure S1: Flow cytometry-gating strategies for time-resolved analysis of the progression of HUVEC infection by *S. aureus*. The gating tree was set as follows: (A) FSC-A/SSC-A to represent the distribution of cells in the light scatter based on size and intracellular composition, respectively, to exclude debris; (B) SSC-H/SSC-A to exclude events that could represent more than one cell; (C): SSC-A/FITC-A to select the infected cell population containing

GFP-expressing bacteria, also shown as a histogram in (D). 20,000 cells were analyzed. The fluorescence distribution of the single infected cell population is shown for the barrier (E) and confluent (F) conditions. To quantify the internalized bacteria released from lysed cell samples, 5000 cells were analyzed from a SSC-A/FITC-A bi-exponential plot (G) to gate GFP-expressing bacteria. (H) Histogram of the ungated population. (I) Histogram of the gated population. (L) Geometric mean fluorescence intensity (GMF) of the host cell population infected with *S. aureus* HG001, D32 or D53 over time for the barrier and confluent conditions, normalized compared to the 2 h p.i. time point.

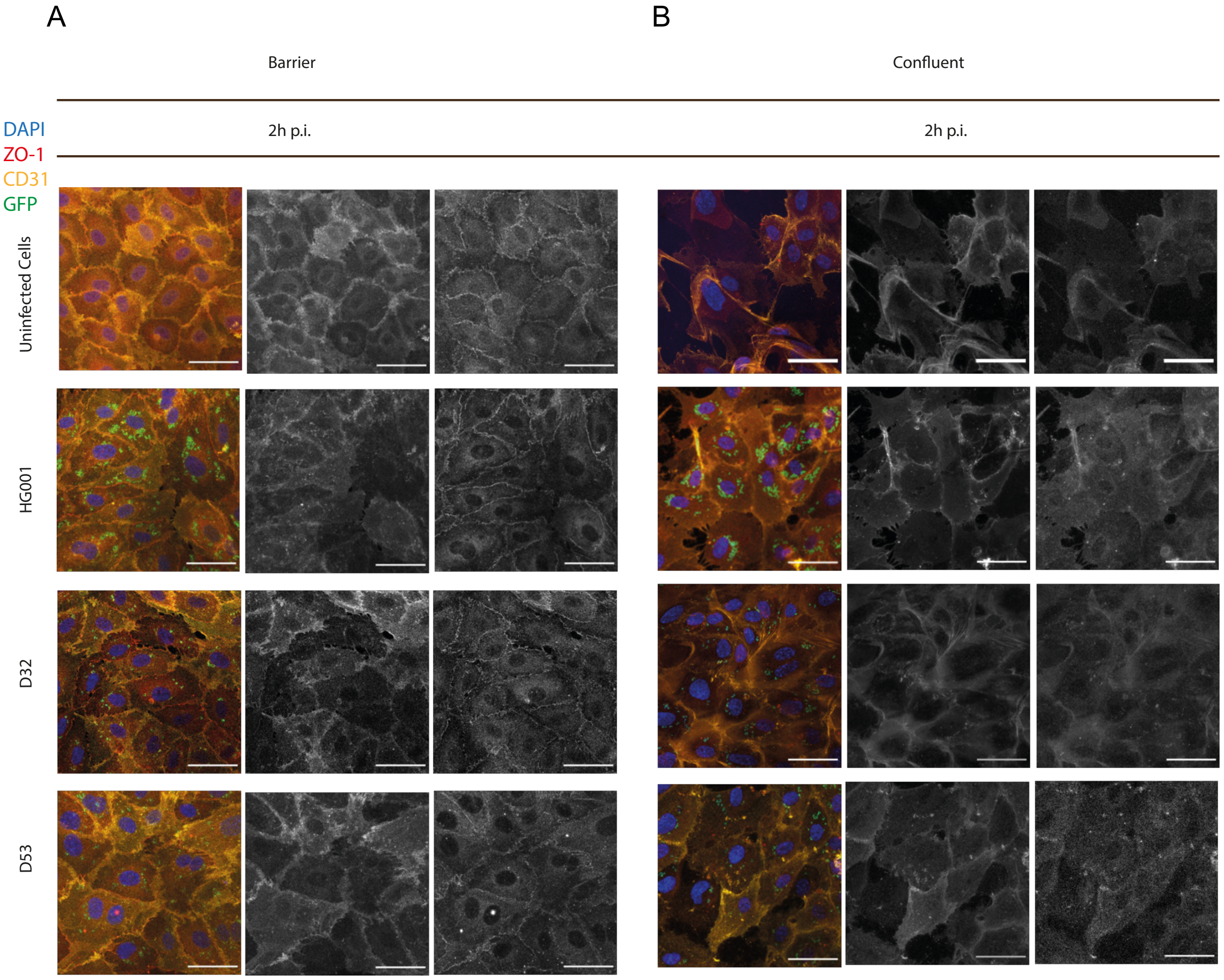

Supplemental Figure S2: Fluorescence microscopy analysis of *S. aureus*-infected HUVEC. The HUVEC were grown in the barrier (A) or confluent conditions (B) and infected with GFP-expressing bacteria of *S. aureus* strains HG001, D32 or D53. Samples collected at 2 h p.i. were immune-stained with specific antibodies to visualize ZO-1 (red in the merged image) and CD31 (yellow in the merged image) as in Figure 2 of the main manuscript. Blue: DAPI-stained nuclei. The micrographs present the maximum pixel value of the Z- stacks of the endothelial layers. Scale bar: 50 µm.

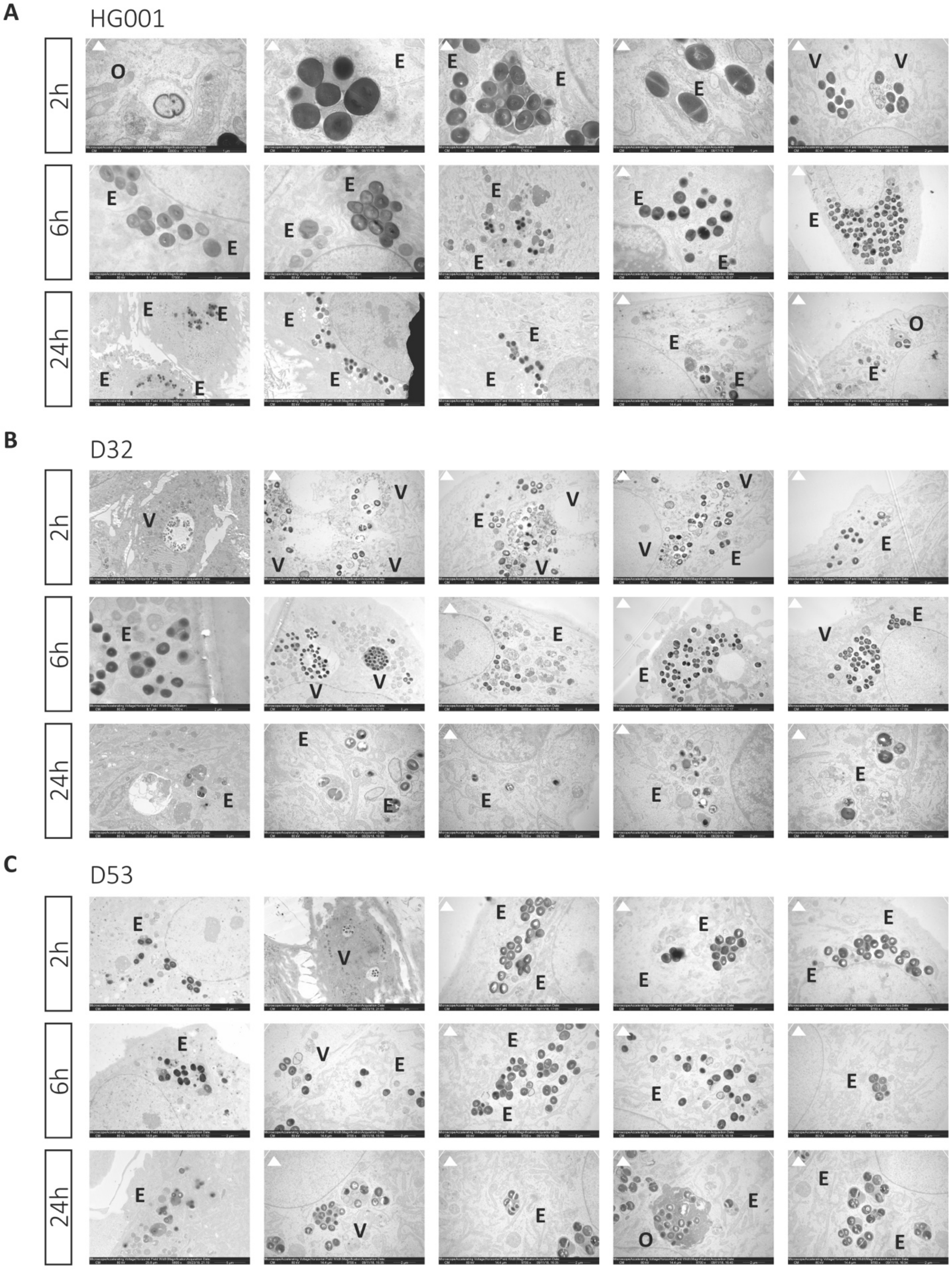

Supplemental Figure S3: Transmission electron microscopy of HUVEC in the barrier or confluent conditions infected with the *S. aureus* strains HG001, D32 or D53. Samples of HUVEC in the barrier or confluent conditions were collected for transmission electron microscopy at 2 h, 6 h and 24 h p.i. with the *S. aureus* strains HG001, D32 or D53 as in Figure 3. For all three strains, the

bacteria remained over time confined to membrane-enclosed compartments, which were either detectable as electron dense compartments (E) of different sizes, or as vacuole-like structures (V) characterized by light intra-compartment staining. No free cytosolic bacteria were detected. O, other structures. Scale bars: 1 µm.

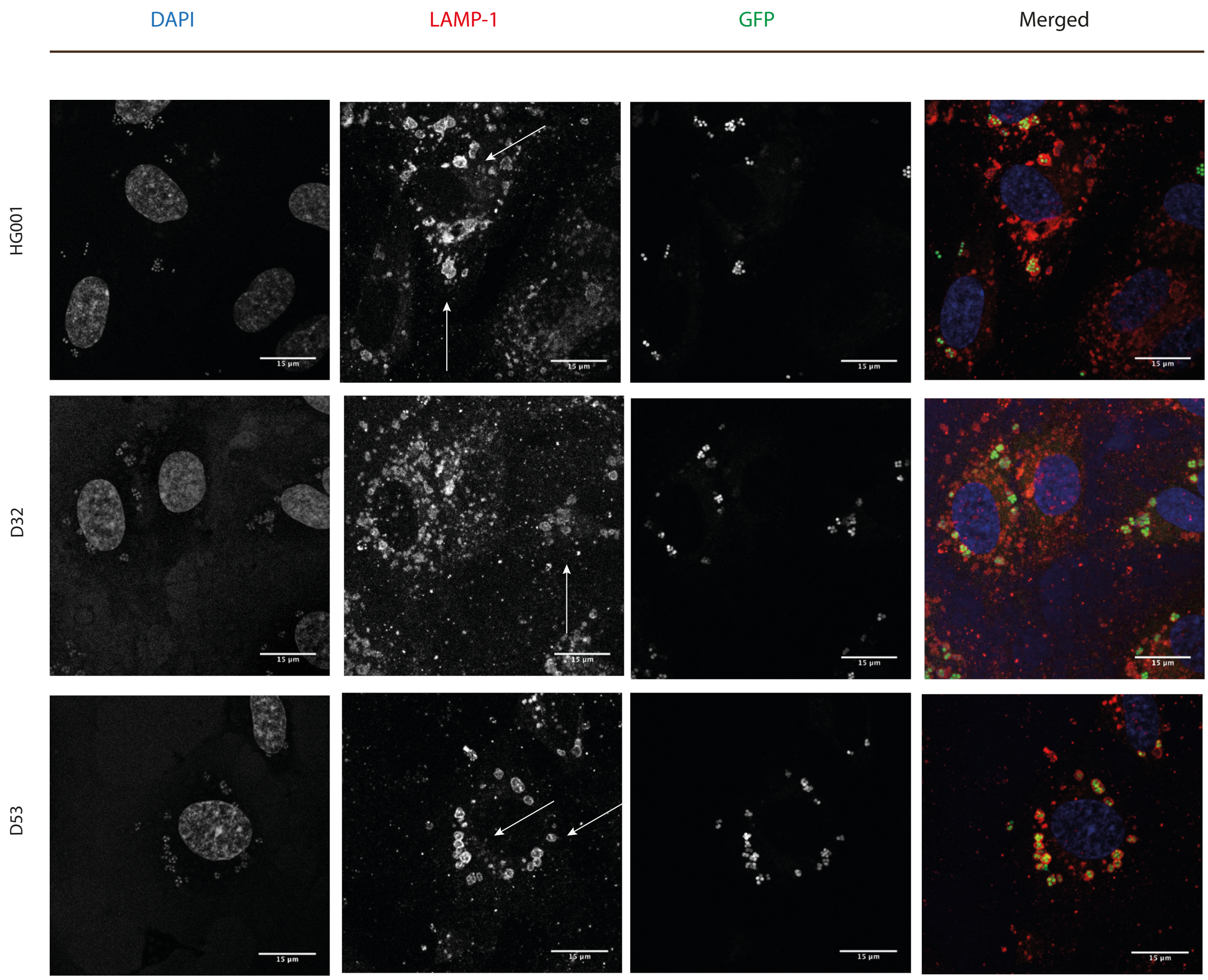

Supplemental Figure S4: Colocalization of internalized *S. aureus* with LAMP-1. Confocal fluorescence microscopy images, showing HUVEC in the barrier condition infected with the *S. aureus* strains HG001, D32 or D53, were recorded at 48 h p.i. as for Figure 4, but are presented at a larger magnification. HUVEC were stained with an anti-LAMP1 (red) antibody. Blue: DAPI-stained nuclei. Green: GFP-expressing bacteria. Arrows indicate examples of bacterial colocalization with LAMP-1.

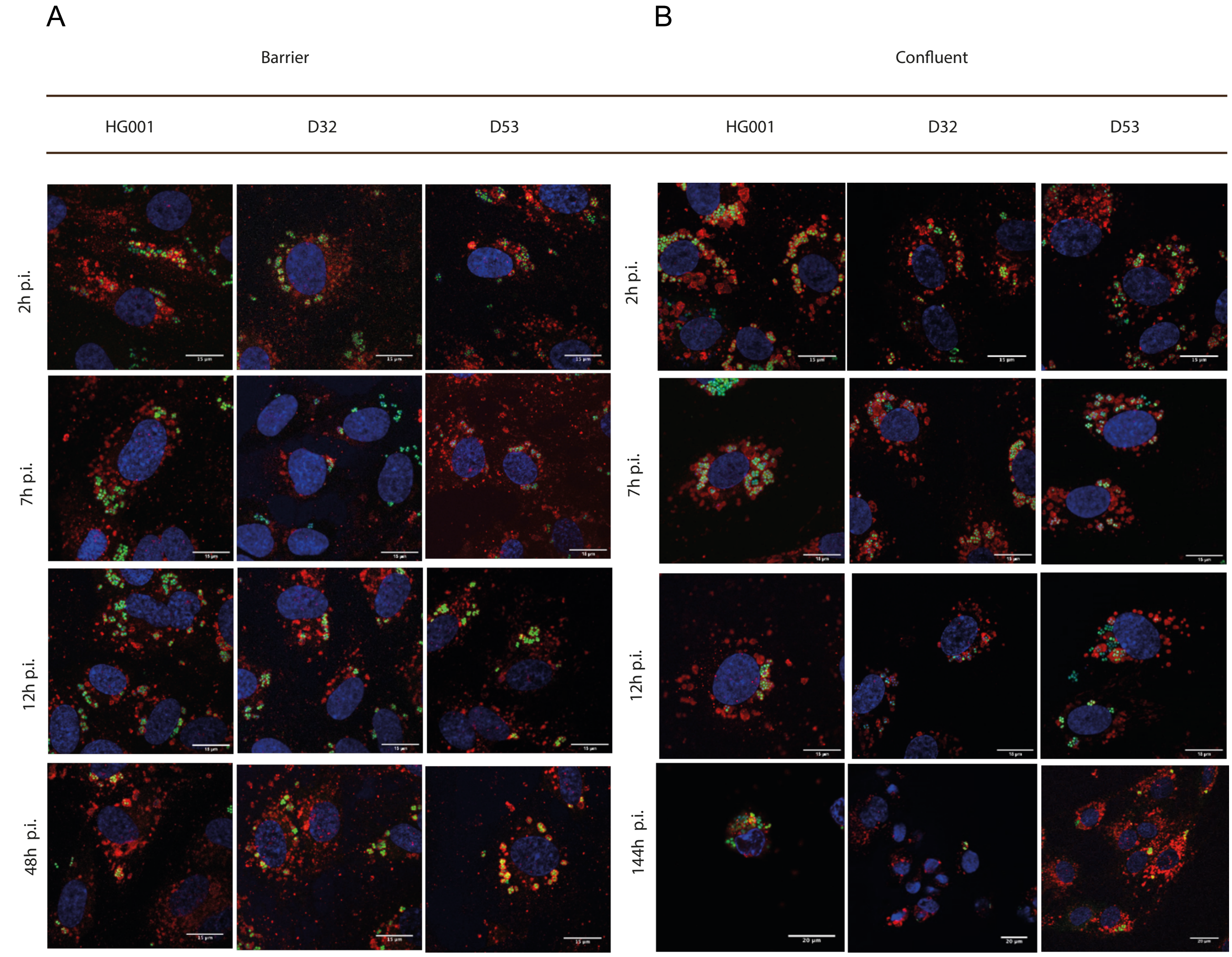

Supplemental Figure S5: Colocalization of HUVEC-internalized *S. aureus* with LAMP-1 over time**.** Confocal fluorescence microscopy images show HUVEC infected with the *S. aureus* strains HG001, D32 or D53 in the barrier (A) or confluent (B) conditions stained with an anti-LAMP1 (red) antibody as in Figure 4. Blue: DAPI-stained nuclei. Green: GFP-expressing bacteria. Co-localization with LAMP-1 was observed at 2 h, 7 h, 12 h, 48 h (barrier model) or 144 h (confluent model) for all tested *S. aureus* strains. Arrows indicate the co-localization.

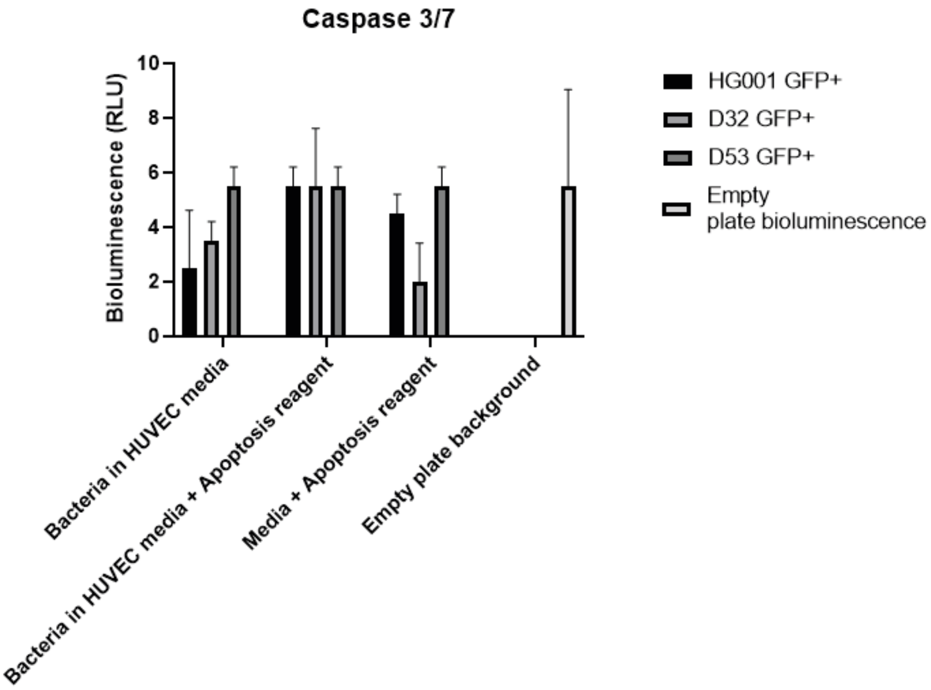

Supplemental Figure S6: *S. aureus* proteases do not cleave caspase 3/7-specific substrates. To verify the possible interference of *S. aureus* proteases with the caspase 3/7 activity assays in Figure 5, samples of *S. aureus* HG001, D32 or D53 were tested using the Caspase-Glo® 3/7 kit. The bioluminescence measurement of the empty plate yielded values, which were comparable to the bioluminescence observed for the bacteria in HUVEC media, bacteria in HUVEC media plus the caspase 3/7 (Apoptosis) reagent, and HUVEC media plus the caspase 3/7 reagent. This shows that no bioluminescence activity is generated by bacterial protease activity.
